# Video Review of Cardiac Arrest: A Scoping Review

**DOI:** 10.1016/j.acepjo.2026.100407

**Published:** 2026-05-05

**Authors:** Micah Wolfsohn, Mohamed Ali, Rahul Ramraj, Timmy Li, Jaclyn Morales, Ghania Haddad, Elizabeth Young, Lance Becker, Daniel Rolston, Daniel Jafari

**Affiliations:** 1 Northwell, New Hyde Park, New York, USA; 2 Donald and Barbara Zucker School of Medicine at Hofstra/Northwell, Hempstead, New York, USA; 3 Touro College of Osteopathic Medicine, New York, New York, USA; 4 North Shore University Hospital, Manhasset, New York, USA

**Keywords:** cardiac arrest, video review, CPR, ROSC

## Abstract

**Objectives:**

As there is no published review of video review use in cardiac arrest (CA) research, we set out to perform a scoping review to describe the demographics, settings, interventions, and outcomes in the literature.

**Methods:**

Following the Preferred Reporting Items for Systematic Reviews and Meta-Analyses framework for scoping reviews, we queried PubMed, Scopus, EMBASE, and Cochrane Library from inception through April 22, 2024, and then updated the query to cover publications through March 7, 2025, including adult CA studies using video-derived data from prehospital, emergency department, or intensive care settings, excluding pediatric and simulation studies. Independent screening and data extraction were both performed by 2 of the reviewers, with a third reviewer and principal investigator resolving discrepancies, respectively. Extracted data encompassed study aims, setting, patient demographics, video review reliability, and detailed information on outcomes, metrics (eg, chest compression fraction), and interventions (eg, intubation).

**Results:**

From 3081 identified publications, 76 were included, with 64.5% being manuscripts. They originated from the USA (48.7%), Asia (31.6%), and Europe (19.7%). Studies were predominantly single center (98.7%), from urban settings (82.9%), with retrospective (47.4%) or prospective (31.6%) observational designs. There was marked heterogeneity in reporting methodologies. The median number of patients enrolled was 71. Interrater reliability was reported in only 12 studies. Common reported patient outcomes included return of spontaneous circulation (39.5%), key metrics such as duration of interruptions (52.6%), and time-to-events (51.3%). Frequently reported interventions included mechanical compression device use (36.8%), defibrillation (34.2%), and intubation (28.9%). Publication volume significantly increased over the last 2 decades.

**Conclusions:**

Video review enables a precise, multidomain assessment of resuscitation performance of CA that conventional data sources cannot provide. Future work should prioritize consensus definitions and establishing minimum reporting standards.

## 1 Introduction

### 1.1 Background

Cardiac arrest (CA) is notoriously difficult to study. Its unpredictable time, course, and settings make real-time observation and data collection challenging.^1^ In addition, CA research is constrained by heterogeneity in patient presentations, treatment environments, and team compositions, all of which can influence outcomes and challenge analysis. Researchers have historically relied on defibrillator monitor data and retrospective chart reviews to assess cardiac arrest care. However, these methods are limited due to recall bias and inconsistent hospital and/or emergency medical services documentation practices, making them suboptimal for evaluating the quality of resuscitative care.^2^^,^^3^

Much of the available CA evidence is derived from data registries that focus on broad outcomes such as return of spontaneous circulation (ROSC) and survival to discharge.^4^ Although invaluable for epidemiologic surveillance, these data sources rarely provide granular insights into the moment-to-moment decision making and performance of resuscitation teams. The dynamic nature of CA, characterized by rapidly changing physiologic states, evolving priorities, and frequent multitasking, means that small deviations in timing, communication, or role clarity can have profound impacts on patient outcomes.^5^

Observational methods, such as in-person shadowing, have been explored but often prove to be impractical considering multiple critical interventions occurring simultaneously in a short period of time.^2^^,^^6^ Simulation-based studies allow for detailed behavioral coding and error analysis, yet findings may not be applicable to the high-pressure, real-world conditions of CA resuscitation.^7^

### 1.2 Importance

CA video review allows for the collection of precise time stamps and vital signs and detailed analysis of team performance, interventions, and patient responses without any of the aforementioned limitations.^8^^,^^9^ Importantly, video review enables assessment of both technical skills (eg, compression quality, defibrillation timing) and nontechnical skills (eg, leadership, closed-loop communication, situational awareness), which have been shown to influence resuscitation quality and outcomes in both simulation and clinical studies.^8^, ^9^, ^10^, ^11^

The field of trauma care has long leveraged video review as a research tool.^12^^,^^13^ In trauma, video review has been credited with identifying latent safety threats, improving protocol adherence, and informing targeted training interventions.^14^, ^15^, ^16^, ^17^ With the growing ubiquity and affordability of video technology, this tool has increasingly been adopted in the study of CA. Nevertheless, the literature remains fragmented, with studies differing in their objectives, recording environments (in-hospital vs prehospital), and approaches to analysis. To date, there is no unified review of the use of video analysis in CA care. The methods, protocols, and applications of video review in this context vary widely. A structured review of the literature is a first step toward a better understanding of use cases, practice environments, and methodological frameworks of CA video review and could open the door to a more unified approach and standardization.

### 1.3 Goals of This Investigation

Here, we conducted a scoping review of CA video reviews, with the aim of characterizing the current landscape of video review in CA for the first time.

## 2 Methods

### 2.1 Study Design and Registration

Following the Preferred Reporting Items for Systematic Reviews and Meta-Analyses extension for scoping review framework^18^ (Supplement A), this scoping review was preregistered on Open Science Framework (https://doi.org/10.17605/OSF.IO/CJ5HM). We queried PubMed, Scopus, EMBASE, and Cochrane Library from inception through April 22, 2024, and then updated the query to cover publications through March 7, 2025.

### 2.2 Search Strategy

Search strategies were formulated using controlled vocabularies and keyword capabilities in each database (detailed search criteria in Supplement B). A complementary keyword search was performed on additional databases (ie, Google Scholar) and within specific journals designated by the research team, as well as a manual search by checking the references of all included articles to identify additional eligible studies.

### 2.3 Selection of Studies

The inclusion criteria were abstracts or manuscripts of adult in-hospital and out-of-hospital cardiac arrest (IHCA and OHCA) patients, with video recording–derived data from prehospital, emergency department, or the intensive care unit. Our exclusion criteria were pediatric studies, simulation video review studies, and patient/family education video studies. Duplicate abstracts of included manuscripts were removed after written confirmation by authors.

### 2.4 Data Extraction and Synthesis

A team of reviewers (TL, RR, DR, JM, MA, MW) was recruited and trained by the principal investigator (DJ). Each study abstract was screened by 2 investigators (TL, RR, DR, JM, MA, MW) independently for eligibility, and disagreements were resolved through a third reviewer (DJ, MA, MW) with the use of the Covidence platform(Veritas Health Innovation, Melbourne).^19^ In the data extraction phase, manuscripts of studies were reviewed by 2 of the investigators(TL, RR, DR, JM, MA)independently, and data were recorded on the Covidence platform. The principal investigator (DJ)then adjudicated discrepancies in the data extractions for each study.

The aim of each study was recorded verbatim from each publication. We also characterized the studies by single center and multicenter. If not explicitly stated, we assessed the text to discern where the data were collected. For prehospital studies, we categorized single-agency studies as single center and multiagency studies as multicenter. We screened the studies for explicitly stated inclusion and exclusion criteria. Inclusion criteria were chosen from a predetermined list of options, whereas the exclusion was recorded as free text by the reviewer.

The geographical setting of the study was also characterized as either urban, suburban, or rural. To determine this, we looked at the medical center description on the center’s website. If this was not available or applicable, we used Google Maps and publicly available population data in their respective catchment areas to categorize them.

In addition, we recorded descriptive statistics such as mean age, sex, estimated downtime, initial rhythm, body mass index, and the duration of the study. Characteristics not reported in the studies were recorded as “missing”. The reporting of interrater reliability between video reviewers was also recorded, as well as relevant statistics.

We organized the data collected by each study into patient-oriented outcomes, metrics, and interventions. Patient-oriented outcomes included ROSC, survival to admission, survival to discharge, and neurologic recovery. Metrics comprised duration of interruptions, time to events, quality of chest compressions, and chest compression fraction. For interventions, we tracked the rates of reporting of mechanical chest compression device (MCCD) use, defibrillation, intubation, ultrasound use, epinephrine administration, other medication administration, and extracorporeal membrane oxygenation (ECMO) cannulation. We also recorded the stated endpoints of the included studies and characterized them as primary, secondary, and reported but not ranked.

## 3 Results

**Figure 1:**
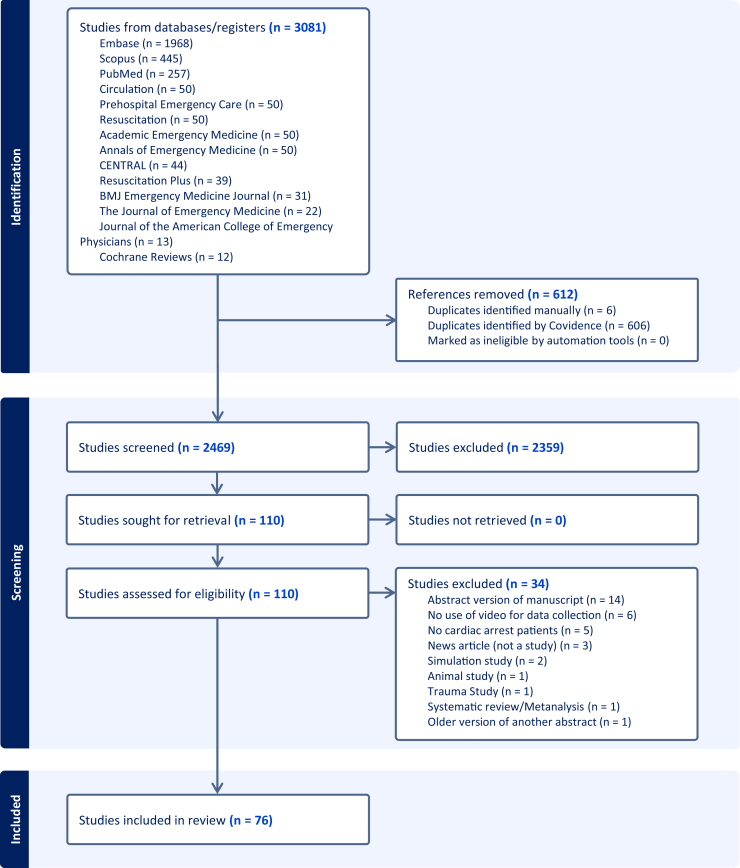
PRISMA flow diagram of the study selection process. The flow diagram depicts the results of the systematic search and screening process conducted in Covidence. A total of 3081 records were identified across databases and registries. After the removal of 611, 2470 studies were screened. Of these, 2359 were excluded, and 111 were retrieved for text review. Following the eligibility assessment, 35 articles were excluded for reasons such as nonhuman subjects, no cardiac arrest patients, and not being a study. Ultimately, 76 studies met eligibility criteria and were included in the review. PRISMA, Preferred Reporting Items for Systematic Reviews and Meta-Analyses.

A total of 3081 publications were identified through the initial search. After removing 611 duplicates, 2470 study titles were screened by study team members, of which 2359 were excluded at title and abstract screening for failing to meet inclusion criteria. An additional 35 studies were excluded for various reasons after screening abstracts, leaving 76 publications for analysis (Fig 1). The Cohen’s Kappa between reviewers ranged from 0.64 to 0.78, with a mean of 0.69.

**Figure 2:**
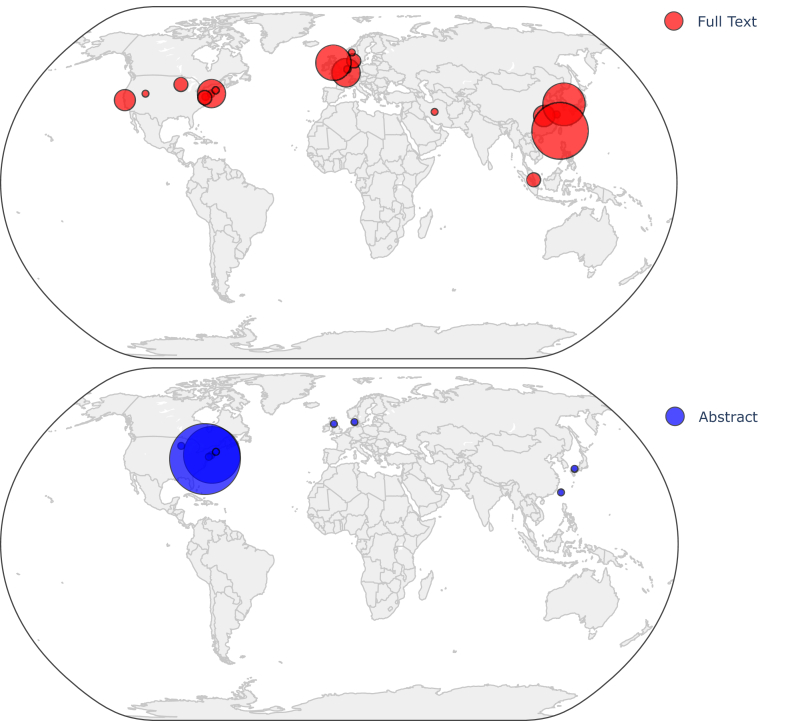
Geographic distribution of included studies. World maps displaying the geographic origins of studies included in this review. The top panel shows studies available as manuscripts (red), whereas the bottom panel shows studies available only as abstracts (blue). Circle size corresponds to the relative number of studies from each location.

Of the 76 included manuscripts and abstracts, 37 (48.7%) were from the USA, 24 (31.6%) from Asia, and 15 (19.7%) from Europe (Fig 2); 27 (35.5%) were abstracts, and 49 (64.5%) were manuscripts. All but 1 were single-center studies, and 83% (n = 63) were performed in urban settings (Table 1). The most common study design was a retrospective observational study (n = 36), followed by prospective observational studies (n = 24). Three studies were interventional, in a pre-post fashion.^20^, ^21^, ^22^ The remainder was a mix of various other study designs, case series, and publication correspondences (Table S1).

**Table 1:** Characteristics of the included publications: this table summarizes the distribution of studies included in the review, categorized by study design (single center vs multicenter), geographic region (United States, Asia, and Europe), and setting (urban, suburban, and rural).

Characteristic	Abstracts (n = 27)	Manuscripts (n = 49)	All publications (n = 76)
Study type			
Single center	27 (100%)	48 (97.9%)	75 (98.7%)
Multicenter	0	1 (2.0%)	1 (1.3%)
Geography			
United States	22 (81.5%)	15 (30.6%)	37 (48.7%)
Asia	3 (11.1%)	21 (42.9%)	24 (31.6%)
Europe	2 (7.4%)	13 (26.5%)	15 (19.7%)
Setting			
Urban	23 (85.2%)	45 (91.8%)	68 (89.5%)
Suburban	4 (14.8%)	1 (2.0%)	5 (6.6%)
Rural	0	1 (2.0%)	1 (1.3%)
Study design			
Retrospective observational	15 (55.6%)	22 (44.9%)	37 (48.7%)
Prospective observational	11 (40.7%)	15 (30.6%)	26 (34.2%)
Before and after	0	4 (8.2%)	4 (5.3%)
Other	1 (3.7%)	8 (16.3%)	9 (11.8%)

Data are presented for abstracts, manuscripts, and all publications. Counts are shown with corresponding percentages relative to the total number within each column.

A total of 56 studies (21 abstracts and 35 manuscripts) specified primary endpoints, and 26 studies (4 abstracts, 22 manuscripts) specified secondary endpoints. Some studies specified both primary and secondary endpoints, whereas others did not specify their endpoints as either (4 abstracts, 9 manuscripts). Of the stated primary endpoints, the most common were time-to-events (n = 11, 19.6%), chest compression fraction (n = 10, 17.9%), and ROSC (n = 4, 7.1%).

Interrater reliability was reported in 12 studies (1 abstract, 11 manuscripts). Of these, interclass correlation coefficients were reported in 7 studies (all manuscripts), whereas a Kappa statistic was reported in 4 studies (all manuscripts), and a Spearman Rho in 1 study (an abstract).

A majority of studies did not report whether they periodically deleted the recordings (only 16 manuscripts did report). Of those which reported their deletion policy, there existed a wide range from 3 days to 1 year. Two studies deleted videos after analysis, and 1 study deleted videos on an overwriting basis (once available storage had been expended).

There was also significant heterogeneity in the hardware and software platforms used for video recording, with 32 (42.1%) of studies reporting the technology or platform used in recording the CA event (4 [14.8%] abstracts, 28 [57.1%] manuscripts) (Table S2). The most commonly reported camera was the GoPro HD Hero 4 (used in 4 studies). Camera mounting solutions also varied and included body-worn, cart-based, in-hospital fixed, and in-ambulance fixed.

The median number of patients included in the analyses was 71 (IQR: 35-136) (Table S3); 39 of the publications included only OHCA; 7 included a mix of OHCA and IHCA, and 1 (conducted in an intensive care unit setting) included only IHCA cases. The remainder of the publications did not include CA location in their stated inclusion criteria. Eleven (14.5%) publications were reporting exclusively on resuscitative efforts in the prehospital environment. The most common inclusion criteria were cardiopulmonary resuscitation (CPR) in the emergency department (n = 47), OHCA (n = 46), availability of video for review (n = 37), and IHCA (n = 7), whereas the most common exclusion criteria were traumatic CA (n = 14); 73 (97.4%) studies reported their inclusion criteria, whereas 42 (55.3%) reported their exclusion criteria. Studies frequently had >1 inclusion or exclusion criterion, which is reported in Table 2.

**Table 2:** Inclusion and exclusion criteria of studies included in the review.

Inclusion criteria			
	Abstracts (n = 27)	Manuscripts (n = 49)	All publications (n = 76)
Adult patients	6 (22.2%)	36 (73.5%)	42 (55.3%)
Out-of-hospital CA	8 (29.6%)	25 (51.0%)	33 (43.4%)
Emergency department	13 (48.1%)	21 (42.9%)	34 (44.7%)
Video available	12 (44.4%)	18 (36.7%)	30 (39.5%)
Other	9 (33.3%)	19 (38.8%)	28 (36.8%)

Exclusion criteria			
	Abstracts (n = 27)	Manuscripts (n = 49)	All publications (n = 76)
Traumatic arrest	3 (11.1%)	11 (22.4%)	14 (18.4%)
Incomplete/poor video recording	2 (7.4%)	9 (18.4%)	11 (14.5%)
ROSC prior to another event	0 (0.0%)	8 (16.3%)	8 (10.5%)
Pediatric patients	1 (3.7%)	5 (10.2%)	6 (7.9%)
DNR/no resuscitation attempted	0 (0.0%)	5 (10.2%)	5 (6.6%)
Signs of irreversible death	0 (0.0%)	2 (4.1%)	2 (2.6%)
MCCD failure	0 (0.0%)	2 (4.1%)	2 (2.6%)

CA, cardiac arrest; DNR, do not resuscitate; MCCD, mechanical chest compression device; ROSC, return of spontaneous circulation.

Data on patient outcomes were reported in 36 studies (7 abstracts, 29 manuscripts), with ROSC being the most common (n = 30, 39.5%), followed by survival to discharge (n = 21, 27.6%), survival to admission (n = 16, 21.1%), and neurologic recovery (n = 7, 9.2%). Interventions were reported in 57 studies (14 abstracts, 43 manuscripts), and metrics were reported in 64 studies (22 abstracts, 42 manuscripts). The most reported interventions were MCCD use (n = 28, 36.8%), defibrillation (n = 26, 34.2%), and intubation (n = 22, 28.9%). Duration of interruptions, quality of chest compressions, and chest compression fraction were reported in 40 (52.6%), 20 (26.3%), and 17 (22.4%), respectively. Time-to-events were reported in 39 (51.3%) publications. Initial rhythms were reported in 35 (46.1%) of included publications, with the most common in each being nonshockable rhythms. Table 3 provides a breakdown of specific rates of reported outcomes, interventions, and metrics broken down by publication type (manuscript vs abstract).

**Table 3:** Summary of the frequency of reporting of outcomes, interventions, and metrics in abstracts, manuscripts, and all publications combined.

Reported data	Abstract (n = 27)	Manuscripts (n = 49)	All publications (n = 76)
Patient outcomes			
ROSC	6 (22.2%)	24 (49.0%)	30 (39.5%)
Survival to hospital discharge	3 (11.1%)	18 (36.7%)	21 (27.6%)
Survival to hospital admission	3 (11.1%)	13 (26.5%)	16 (21.1%)
Neurologic recovery	0 (0.0%)	7 (14.3%)	7 (9.2%)
Interventions			
MCCD	6 (22.2%)	22 (44.9%)	28 (36.8%)
Defibrillation	4 (14.8%)	22 (44.9%)	26 (34.2%)
Intubation	2 (7.4%)	20 (40.8%)	22 (28.9%)
Ultrasound	5 (18.5%)	11 (22.4%)	16 (21.1%)
Epinephrine administration	0 (0.0%)	7 (14.3%)	7 (9.2%)
Other medication administration	0 (0.0%)	6 (12.2%)	6 (7.9%)
ECMO	0 (0.0%)	6 (12.2%)	6 (7.9%)
Metrics			
Duration of interruptions	14 (51.9%)	26 (53.1%)	40 (52.6%)
Time-to-events	13 (48.1%)	26 (53.1%)	39 (51.3%)
Quality of chest compressions	5 (18.5%)	15 (30.6%)	20 (26.3%)
Chest compression fraction	7 (25.9%)	10 (20.4%)	17 (22.4%)

Outcomes included ROSC, survival to hospital admission, survival to hospital discharge, and neurologic recovery. Interventions assessed included defibrillation, epinephrine administration, other medication administration, MCCD, intubation, ultrasound, and ECMO. Metrics reported included time-to-events, chest compression fraction, quality of chest compressions, and duration of interruptions. Counts and percentages are shown by publication type.

ECMO, extracorporeal membrane oxygenation; MCCD, mechanical chest compression device; ROSC, return of spontaneous circulation.

**Figure 3:**
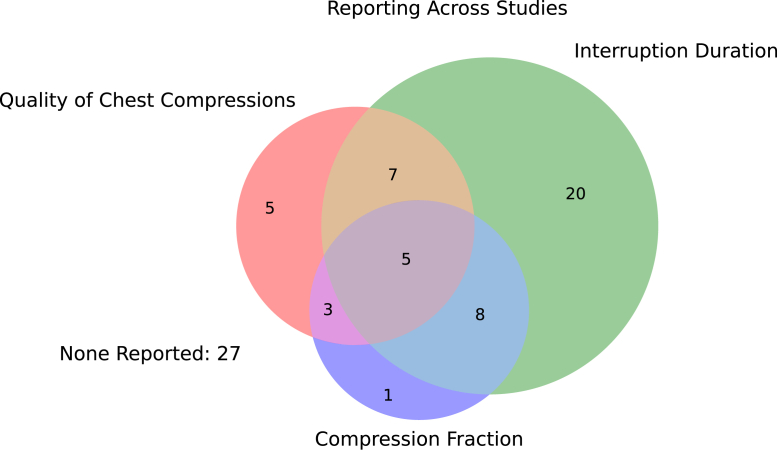
The overlap of reporting of quality of chest compressions, interruption duration, and chest compression fraction across all studies. None of these metrics were reported in 27 (35.5%) of the 76 publications.

**Figure 4:**
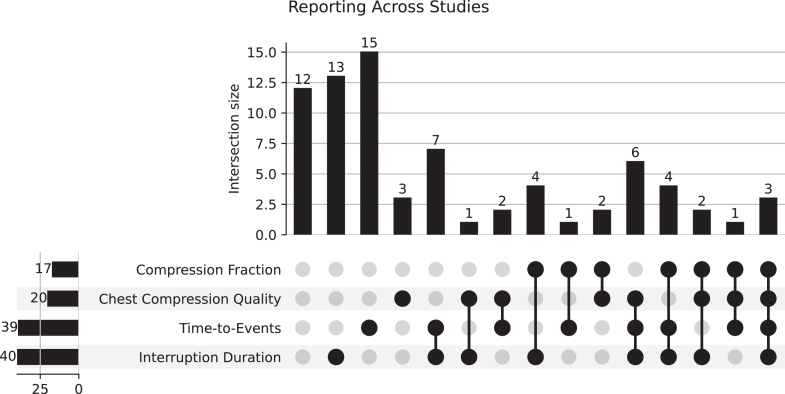
An upset plot showing the reporting of outcomes across all included studies. Each vertical bar represents the number of studies reporting a specific combination of outcomes, whereas the connected dots below indicate which interventions were included in that combination. Horizontal bars show the total number of studies reporting each outcome overall. The plot displays the significant heterogeneity in reported interventions across the publications in our review.

**Figure 5:**
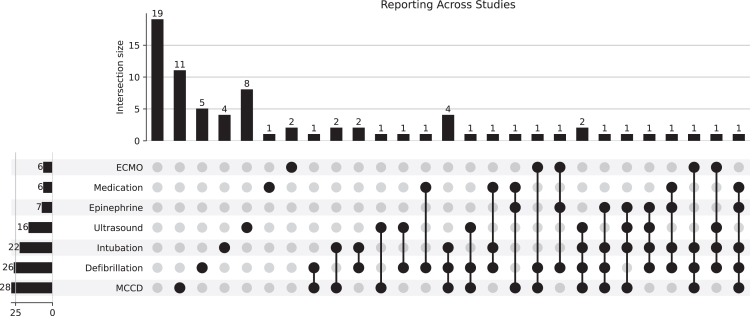
An upset plot showing the reporting of interventions across all included studies. Each vertical bar represents the number of studies reporting a specific combination of interventions, whereas the connected dots below indicate which interventions were included in that combination. Horizontal bars show the total number of studies reporting each intervention overall. The plot displays the significant heterogeneity in reported interventions across the publications in our review.

Many studies simultaneously reported multiple outcomes and/or interventions. To better demonstrate this, we presented a selection of common metrics reported using a Venn diagram (Fig 3), as well as upset plots (Figs 4 and 5) to portray the overlap.

**Figure 6:**
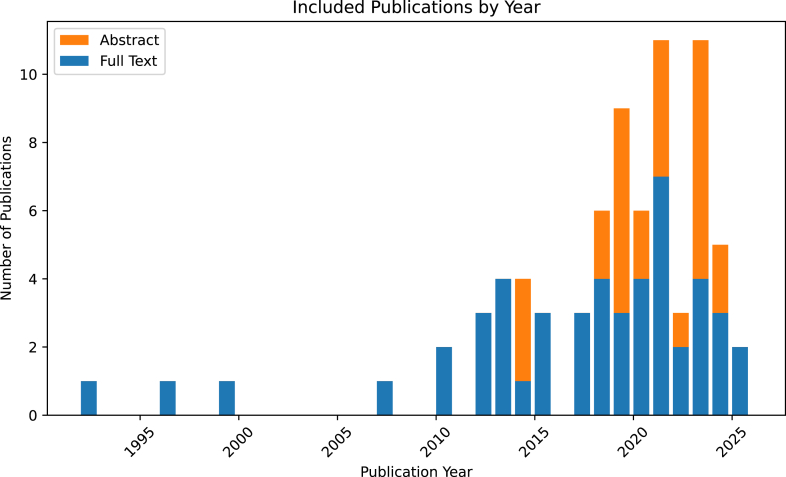
Breakdown of the years of publication of manuscripts and abstracts included in this review.

Since the first published report of the use of video review in CA in 1992,^23^ we found a steady rise with a substantial increase in publications over the past 2 decades. Thirteen of the included publications were from 2010 to 2014, 21 were from 2015 to 2019, and 36 were from 2020 to 2024 (Fig 6).

**Figure 7:**
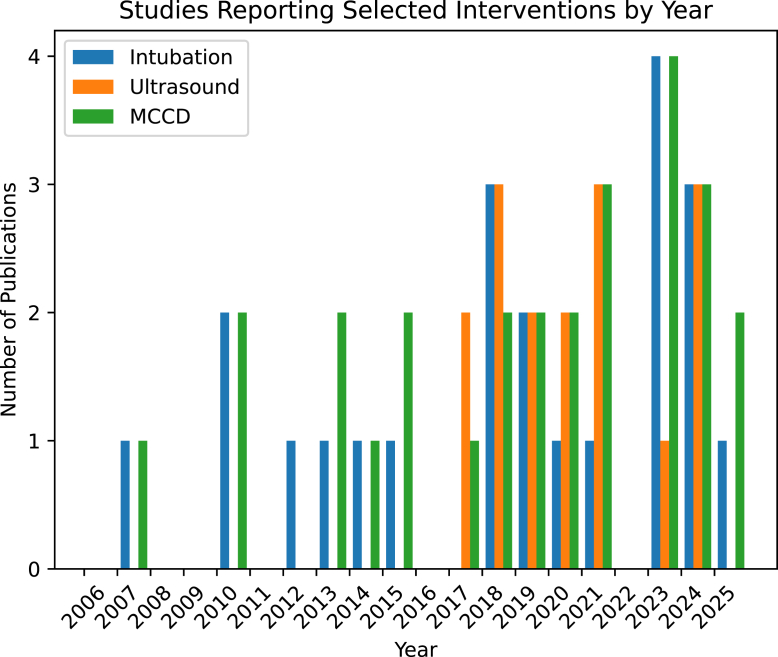
Reporting of specified interventions by publication year.

We charted the number of studies reporting intubation, ultrasound, and MCCD use by year of publication (Fig 7). Intubation and MCCD use have been reported sporadically since 2007, whereas the reporting of ultrasound use in CA video review publications began in 2017, but has shown consistency since.

## 4 Limitations

This scoping review has several methodological limitations that merit consideration. We did not impose standardized definitions of outcomes such as ROSC or neurologic recovery, instead relying on each study’s reported definitions, which may limit direct comparability across studies. We did not perform a critical appraisal or quality assessment of included sources of evidence and thus cannot comment on the methodological rigor or risk of bias within individual studies. Our inclusion of abstracts may have introduced additional limitations, as these publications contain limited methodological detail. Furthermore, we did not contact study investigators to obtain unpublished data or request missing information.

Our approach to categorizing study characteristics (such as urban vs suburban vs rural and single vs multicenter) also involved subjective interpretation when not explicitly mentioned by studies and may have introduced misclassification. The heterogeneity in study aims and designs also posed challenges for standardized data extraction, and the underreporting of key methodological details—such as technology platforms and interrater reliability—further limited our ability to assess methodological trends. Finally, the descriptive nature of our synthesis precluded quantitative pooling of outcomes or meta-analysis due to substantial heterogeneity in study designs, populations, and reported measures.

## 5 Discussion

CA research has long been constrained by the lack of temporal precision and behavioral granularity. Although previous reviews discussed video review as one among many technologies,^24^ this is, to our knowledge, the first to exclusively map the use of video review in CA research, identifying video review as a methodology uniquely suited to address these gaps. We found that video review enables 3 capabilities that distinguish it from conventional approaches: precise measurement of time-sensitive processes, capture of data that are otherwise impossible to collect, and simultaneous evaluation of multiple performance domains.

The most frequently reported metrics in our review—duration of interruptions (52.6%) and time-to-events (51.3%)—illustrate the core strength of this technology. These time-critical variables are central to resuscitation quality, as delays in defibrillation and prolonged chest compression pauses have been directly linked to worse outcomes.^25^^,^^26^ Manual timekeeping during resuscitation is unreliable, and defibrillator monitor data provide only indirect measures of team performance.^27^^,^^28^ Video review allows frame-by-frame analysis of these intervals, offering a level of precision that other methods cannot match.

Beyond timing data, video review permits evaluation of variables that no other methodology can capture at all. Compression location over the chest, for example, can only be assessed visually.^29^ Collecting data simultaneously from multiple proprietary devices (eg, defibrillator, bedside monitor, ventilator, ultrasound machines) is another example.^30^ Similarly, nontechnical skills such as team leader performance and communication patterns require direct observation of team dynamics that chart review and monitor data simply do not provide.

Our finding that the majority of included studies reported multiple categories of video-derived data simultaneously (Figure 3, Figure 4, Figure 5) confirms that investigators are leveraging this multidomain capability, extracting both technical (ie, time-to-event) and nontechnical (team performance) data from the same recordings.

Randomized controlled trials in CA are complex, costly, and logistically challenging, in part because the outcomes most likely to demonstrate an effect—such as survival to discharge and neurologic recovery—require large sample sizes and long follow-up periods where many confounders can alter outcomes.^31^ By enabling precise measurement of surrogate markers such as time-to-defibrillation, chest compression fraction, and interruption duration, video-based studies can generate preliminary evidence on whether an intervention improves the process of resuscitation before large-scale trials. This “staging ground” role is evident in 3 preinterventional and postinterventional studies we identified.^20^, ^21^, ^22^ If more widely adopted, video review could accelerate the pipeline from quality improvement observation to clinical trial by identifying interventions which merit investment in larger studies.

However, realizing this potential requires addressing the methodological heterogeneity we identified. Our results revealed variation in virtually every aspect of how video review studies are conducted: camera hardware and mounting (body-worn, cart-based, fixed overhead), recording storage and deletion policies (ranging from 3 days to 1 year), and definitions of events and outcomes. Only 42.1% of studies reported the technology platform used, and only 12 studies (15.8%) reported interrater reliability—a notable gap for a methodology that depends on human interpretation of visual data. This undermines the ability to compare findings across studies or build a cumulative evidence base. If 2 studies both report “time-to-defibrillation” but define the start time differently—one from the moment of collapse and the other from the moment of start of video recording—their results are not comparable, regardless of how precisely each was measured.

We identified 2 priorities that would most directly improve video review. First, consensus definitions for the most commonly reported video-derived metrics—particularly interruption duration, time-to-event intervals, and chest compression quality parameters—would enable cross-study comparisons.^32^ These were the most frequently reported data points in our review, and standardizing their definitions would yield the greatest return. Second, routine reporting of interrater reliability should become an expected methodological standard, given that only a small minority of studies currently do so. Lastly, we believe the integration of artificial intelligence to analyze and timestamp events in the recordings^33^ would remove the rate-limiting step of video analysis by human experts and increase adoption of this technology.

In summary, this scoping review demonstrates that video review occupies a unique and currently underutilized methodological niche in CA research—one that enables precise, multidomain assessment of resuscitation performance that conventional data sources cannot provide. Prioritizing consensus definitions for commonly reported metrics and establishing minimum reporting standards for video review methodology are of paramount importance for wider adoption.

## Author Contributions

Micah Wolfsohn: Software, formal analysis, investigation, writing—original draft, visualization

Mohamed Ali: Investigation, resources

Dr Rahul Ramraj: Conceptualization, writing—review and editing

Dr Timmy Li: Conceptualization, writing—review and editing

Jaclyn Morales: Methodology, resources, validation

Dr Ghania Haddad: Conceptualization

Elizabeth Young: Conceptualization

Dr Lance Becker: Supervision, writing—review and editing

Dr Daniel Rolston: Writing—review and editing, methodology, project administration

Dr Daniel Jafari: Supervision, writing—original draft, project administration, conceptualization, methodology, validation

## Funding and Support

By *JACEP Open* policy, all authors are required to disclose any and all commercial, financial, and other relationships in any way related to the subject of this article as per ICMJE conflict of interest guidelines (see www.icmje.org). The authors have stated that no such relationships exist.

## Conflict of Interest

Dr Daniel Rolston reports receiving a research grant from Flosonics Medical.

Dr Daniel Jafari reports having received research grants from Theravance Biopharma and the Zoll Foundation in the past.

Dr Timmy Li reports receiving a research grant from the Zoll Foundation.

Dr Ghania Haddad reports receiving a research grant from Laerdal Medical.

Dr Lance Becker reports serving as a consultant for Philips Healthcare.

All other authors declare that they have no conflicts of interest related to this work.
