# Supplementary material for: Video Review of Cardiac Arrest: A Scoping Review
Source: J Am Coll Emerg Physicians Open. 2026 May 5;7(3):100407. doi: 10.1016/j.acepjo.2026.100407 (PMC13158408; doi:10.1016/j.acepjo.2026.100407)
Supplement: Supplementary Tables 1-3 [file mmc1.docx]

Supplement Table 1: Study design

| **Study Design** | **Abstract (n=27)** | **Full Text (n=49)** | **Total (n=76)** |
| --- | --- | --- | --- |
| Retrospective Observational | 15 (55.6%) | 22 (44.9%) | 37 (48.7%) |
| Prospective Observational | 11 (40.7%) | 15 (30.6%) | 26 (34.2%) |
| Before-and-After | 0 | 4 (8.2%) | 4 (5.3%) |
| Letters and Replies | 0 | 3 (6.1%) | 3 (3.9%) |
| Case Report | 1 (3.7%) | 1 (2.0%) | 2 (2.6%) |
| Case Series | 0 | 2 (4.1%) | 2 (2.6%) |
| Cohort Study | 0 | 2 (4.1%) | 2 (2.6%) |

Supplement Table 2. Recording hardware (a) and software (b) reported to be used for cardiac arrest video recording

2a.

| **Hardware** | **Abstract (n=27)** | **Full Text (n=49)** | **All Publications (n=76)** |
| --- | --- | --- | --- |
| GoPro HERO 4 | 0 | 4 (8.2%) | 4 (5.3%) |
| DS-8000 | 0 | 3 (6.1%) | 3 (3.9%) |
| RVT-SD200 Sony transmission device | 1 (3.7%) | 0 | 1 (1.3%) |
| CCTV cameras at the location of the cardiac arrest | 1 (3.7%) | 0 | 1 (1.3%) |
| ICU-based telemedicine video recording system (platform name not specified) | 1 (3.7%) | 0 | 1 (1.3%) |
| DrivePro Body 10 | 0 | 1 (2.0%) | 1 (1.3%) |
| MPCAM Z07 | 0 | 1 (2.0%) | 1 (1.3%) |
| Insta360 ONE R | 0 | 1 (2.0%) | 1 (1.3%) |
| SJCAM A10 | 0 | 1 (2.0%) | 1 (1.3%) |
| Ikegami CV 770-Auto iris 8mm | 0 | 1 (2.0%) | 1 (1.3%) |
| Sony Handycam CCD | 0 | 1 (2.0%) | 1 (1.3%) |
| MagicRadar Cs16 System | 0 | 1 (2.0%) | 1 (1.3%) |

2b.

| **Software** | **Abstract (n=27)** | **Full Text (n=49)** | **All Publications (n=76)** |
| --- | --- | --- | --- |
| Tru-Vision software | 0 | 2 (4.1%) | 2 (2.6%) |
| Avigilon software | 0 | 2 (4.1%) | 2 (2.6%) |
| See-CPR | 1 (3.7%) | 0 | 1 (1.3%) |
| REDCap | 0 | 1 (2.0%) | 1 (1.3%) |
| Insta360 Studio | 0 | 1 (2.0%) | 1 (1.3%) |
| Ocularis | 0 | 1 (2.0%) | 1 (1.3%) |
| TruVision | 0 | 1 (2.0%) | 1 (1.3%) |
| WiseNet | 0 | 1 (2.0%) | 1 (1.3%) |

Supplement Table 3: Full list of abstracts and full texts included, with geopgrahic area, setting, location, study aim, study design, and total number of patients included

| Publication year | Type | Title | Geography | Setting | Location | Aim of Study | Study design | Total Number of Patients |
| --- | --- | --- | --- | --- | --- | --- | --- | --- |
| 2024 | Abstract | Assessment of Transesophageal Echocardiography on Chest Compression Fraction and Clinical Outcome in Patients with Non-Traumatic Out-of-Hospital Cardiac Arrest | Asia | Urban | Chiayi County, Taiwan, TW | To explore the impact of TEE on chest compression fraction (CCF) and patient survival throughout the resuscitation process | Retrospective observational study | 27 |
| 2024 | Abstract | A Video Review-Based Clinical Assessment Tool of Cardiac Arrest Resuscitation Correlates With Higher Chest Compression Fraction | United States | Suburban | Manhasset, NY | To examine if higher clinical assessment scores are associated with higher chest compression fractions | Retrospective observational study | 325 |
| 2023 | Abstract | Team Composition in the Resuscitation Bay: Which Team Members Improve Performance Times During Cardiac Arrest | United States | Suburban | Manhasset, NY | To investigate the association between number of MDs, RNs, and ED technicians involved in the resuscitation and time to critical events (TTCEs). | Retrospective observational study | 192 |
| 2021 | Abstract | Factors associated with time to transesophageal probe placement during cardiac arrest in the emergency department | United States | Urban | Minneapolis, MN | Assessing clinical factors associated with time to transesophageal echocardiography imagine acquisition during cardiac arrest. | Retrospective observational study | 62 |
| 2021 | Abstract | Effect of Routine Bedside Echo and Code Echo Experience on Image Acquisition Time During Cardiac Arrest | United States | Urban | Worcester, MA | To assess the association between echo image acquisition time and experience levels | Prospective observational study | 141 |
| 2020 | Abstract | Dont let the monitor fool you: pulse check variation between shockable and non-shockable rhythms | United States | Urban | Washington, D.C. | To determine if there was a difference in pulse check times between OHCA patients presenting with shockable vs non-shockable rhythms. | Prospective observational study | 97 |
| 2021 | Abstract | CPR Duration and Temporal Characteristics of the Resuscitation | United States | Urban | Washington, D.C. | To examine the relationship between the temporal characteristics of a resuscitation and CPR duration | Prospective observational study | 96 |
| 2021 | Abstract | Duration of Interruptions in Chest Compressions Increases with Increasing Number of Interventions During Cardiac Arrest | United States | Urban | Manhasset, NY | To see if the median duration of interruption increases as the number of interventions increases and that the median duration of an interruption increases if it occurs later in the resuscitation | Retrospective observational study | 122 |
| 2019 | Abstract | Verbalisation of Plans During Out-of-Hospital Cardiac Arrest Resuscitation | Europe | Urban | Edinburgh, UK | To investigate verbal communication during out-of-hospital cardiac arrest (OHCA) for plan verbalisation patterns and possible associations with successful or unsuccessful outcomes. | Retrospective observational study | 10 |
| 2019 | Abstract | Improving Ultrasound use in CPR to Minimize Duration of Pulse Checks | United States | Urban | Washington, DC | To evaluate the effect of a resident-focused educational intervention on the length of pulse checks | Prospective observational study | 47 |
| 2014 | Abstract | Video Analysis of Cardiopulmonary Resuscitation Performance of Ambulance Crews During Transportation | Asia | Urban | Hiroshima, Japan | To evaluate the performance of CPR provided by ambulance crews and reveal reasons for hands off time during CPR | Prospective observational study | 32 |
| 2014 | Abstract | A Novel Video-Based Motion Analysis System to Evaluate Performance of Cardiopulmonary Resuscitation in Ambulance Transport | Asia | Urban | Taipei, Taiwan | To examine the utilization of a reliable video-based CPR motion detection and analysis system to report CPR performance in ambulance transport. | Prospective observational study | 20 |
| 2023 | Abstract | The Bystander Effect: The Influence of Known Bystander CPR and Witnessed Arrest on ED CPR Duration | United States | Urban | Washington, D.C. | To evaluate the relationship between known bystander CPR and witnessed cardiac arrest on physician decision to terminate resuscitation in the ED | Prospective observational study | 71 |
| 2023 | Abstract | When to terminate: Impact of Prehospital CPR on Emergency Department Resuscitations | United States | Urban | Washington, DC | To evaluate the relationship between prehospital emergency medical services (EMS) CPR and emergency department (ED) CPR duration before resuscitation termination. | Prospective observational study | 71 |
| 2014 | Abstract | Using Surveillance video for insight into Out-of-Hospital Cardiac Arrest (OHCA) | Europe | Urban | Copenhagen, Denmark | To combine CCTV and audio recording from the emergency medical dispatcher (EMD) in order to identify the learning possibilities | Case report | 1 |
| 2019 | Abstract | The Need for Public Awareness in the Washington D.C. Community for Witnessed Cardiac Arrest | United States | Urban | Washington, D.C. | To examine the outcomes of out-of-hospital arrest to assess the need for community education | Prospective observational study | 44 |
| 2023 | Abstract | Positioned for Success: A Novel Exploration of Changes to Chest Compressions During Cardiopulmonary Resuscitation and Associated Patient Outcomes | United States | Suburban | Manhasset, NY | Exploring the frequency of chest compressions changes during CPR, characterized these changes, and reported associated patient outcomes | Retrospective observational study | 18 |
| 2019 | Abstract | Chest Compressions Interruptions During Mechanical Chest Compression Device Placement: A Video Review Analysis | United States | Urban | Manhasset, NY | To quantify the total duration of chest compression interruptions during MCCD placement, determine the total duration of time for MCCD placement and describe the frequency of chest compression interruptions during MCCD placement. | Retrospective observational study | 54 |
| 2019 | Abstract | Implementation of a New Emergency Medical Services Handoff Process for Cardiac Arrest Patients | United States | Suburban | Manhasset, NY | To determine whether a new EMS handoff process would reduce 1) time-to-bed transfer, 2) duration of interruptions, and 3) time-to-initial rhythm determination. | Retrospective observational study | 42 |
| 2022 | Abstract | Size doesn't matter: determining association between BMI and Pulse check length in cardiac arrest | United States | Urban | Washington, DC | Evaluate if a larger body habitus would increase pulse check length during CPR | Prospective observational study | 82 |
| 2018 | Abstract | Focused Transthoracic Echocardiography is Associated with increased Chest Compression Interruptions during Cardiopulmonary Resuscitation in the Emergency Department | United States | Urban | Brooklyn, NY | To determine whether focused transthoracic echocardiography (ECHO) use during cardiopulmonary resuscitation (CPR) increased the duration of chest compression interruptions | Retrospective observational study | 210 |
| 2023 | Abstract | Tracheal intubation prior to arrival or cardiac arrest in the emergency department is associated with lower odds of return of spontaneous circulation: a video review registry study | United States | Urban | Manhasset, NY | Determine if intubation prior to arrival to emergency department (ED) or prior to CA is associated with shorter time to critical interventions, including time to rhythm check, defibrillation, and higher rates of return of spontaneous circulation (ROSC) | Retrospective observational study | 324 |
| 2023 | Abstract | Time and Day of Cardiac Arrest Presentation to the Emergency Department Associations With Time to Critical Interventions and Outcomes: A Video Review Study | United States | Urban | Manhasset, NY | To analyze whether time to rhythm analysis, first defibrillation of a shockable rhythm, and intubation differed between daytime or night time and weekday or weekends. | Retrospective observational study | 283 |
| 2023 | Abstract | Lost in Translation: Video Review to Assess EMS Handoff in Out-of-Hospital Cardiac Arrest | United States | Urban | Washington, D.C. | To use video review to assess quality of EMS handoff in out of hospital cardiac arrest. | Prospective observational study | 35 |
| 2020 | Abstract | Age Is the Only Factor That Affects Survival to Hospital Admission in Video-Reviewed Out- of-Hospital Cardiac Arrest Resuscitations | United States | Urban | Washington, DC | To investigate factors associated with survival to hospital admission | Retrospective observational study | 96 |
| 2019 | Abstract | Pulse Check Improvement Through Video Analysis and Feedback | United States | Urban | Washington, D.C. | To analyze the efficacy of a quality video review-based educational intervention in minimizing pulse check times and maximizing compression ratio for all out-of-hospital cardiac arrests | Retrospective observational study | |
| 2018 | Abstract | Leveraging Telemedicine for QI Video Review of Critical ICU events: A Novel Educational Tool | United States | Urban | Philadelphia, PA | To analyze the events triggering recording of bedside events and report performance issues identified that may then be used to inform ICU quality improvement (QI) efforts | Retrospective observational study | 136 |
| 2025 | Full Text | Bystander Cardiopulmonary Resuscitation and Outcomes of Mass Cardiac Arrests Caused by a Crowd Crush | Asia | Urban | Seoul, South Korea | To investigate how bystander CPR was administered to patients experiencing cardiac arrest during a crowd crush event, evaluate the extent of airway support provided, and analyze the outcomes of these mass cardiac arrests (MCAs). | Case series | 29 |
| 2025 | Full Text | Left of Sternum Compressions Are Associated With Higher Systolic Blood Pressure Than Lower Half of Sternum Compressions in Cardiac Arrest | United States | Suburban | Manhasset, NY | To compare the highest arterial line systolic blood pressure (SBP) and end-tidal CO2 (ETCO2) during lower-half-of-sternum chest compressions versus those left-of-sternum. | Retrospective observational study | 24 |
| 2024 | Full Text | Development and Evaluation of a Novel Resuscitation Teamwork Model for Out-of- Hospital Cardiac Arrest in the Emergency Department | Asia | Urban | Taipei, Taiwan | To implement the Airway-Circulation-Leadership-Support (A-C-L-S) teamwork mode in ED settings to improve resuscitation team performance, particularly in adherence to protocols, timeliness of CPR task execution, and overall CPR quality. | Retrospective observational study | 104 |
| 2017 | Full Text | Chest Compression Fraction in Ambulance While Transporting Patients With Out-of-Hospital Cardiac Arrest to the Hospital in Rural Taiwan | Asia | Rural | Nantou County, Taiwan | To explore the actual prehospital state of chest compressions in an ambulance travelling to the hospital‚Äôs emergency department (ED) in rural Taiwan. | Retrospective observational study | 102 |
| 2024 | Full Text | The Association of Time to Key Prehospital Interventions Recorded by EMT-worn Video Devices and Sustained Return of Spontaneous Circulation in Out-of-Hospital Cardiac Arrests | Asia | Urban | Hsinchu City, Taiwan | To investigate the relationship between 6 quality indices as identified through video review and patient outcomes | Retrospective observational study | 745 |
| 2024 | Full Text | Success of focused transthoracic echocardiography locations for cardiac visualization during cardiac arrest: A video-review analysis | United States | Urban | Manhasset, NY | Determine if there was a difference in success of cardiac visualization by focused transthoracic echocardiography (TTE) location (subxiphoid, parasternal or apical) during chest compression interruptions among cardiac arrest patients. Secondarily, we sought to determine whether there were differences in chest compression interruption times with the focused TTE locations | Retrospective observational study | 136 |
| 2021 | Full Text | Echocardiographic Pre-Pause Imaging and Identifying the Acoustic Window During CPR Reduces CPR Pause Time during ACLS - A Prospective Cohort Study | United States | Urban | Worcester, MA | To describe the effect of pre-pause imaging on CPR pause length in out of hospital cardiac arrest patients. | Cohort study | 145 |
| 2013 | Full Text | Improving the quality of cardiopulmonary resuscitation by training dedicated cardiac arrest teams incorporating a mechanical load-distributing device at the emergency department | Asia | Urban | Singapore, Singapore | To determine if implementing cardiac arrest teams trained with a ‚Äòpit-crew‚Äô protocol incorporating a load-distributing band mechanical CPR device (Autopulse) improves the quality of CPR, as determined by no-flow ratio (NFR) in the first 10 minutes of resuscitation. | Before-and-after | 248 |
| 2019 | Full Text | Transesophageal Echocardiography During Cardiopulmonary Resuscitation Is Associated With Shorter Compression Pauses Compared With Transthoracic Echocardiography | United States | Urban | Salt Lake City, Utah | To assess whether the use of transesophageal echocardiography resulted in decreased pause duration compared with transthoracic echocardiography or manual pulse checks. | Retrospective observational study | 25 |
| 2023 | Full Text | A Novel Assessment Using a Panoramic Video Camera of Resuscitation Quality in Patients following Out-of-Hospital Cardiac Arrest | Asia | Urban | New Taipei City, Taiwan | To discuss the strengths and drawbacks of the chest pads, single-angle video camera (SAC), and panoramic video camera for assessing CPR quality and team performance in an out-of-hospital setting | Case report | 1 |
| 2023 | Full Text | First attempt success with continued versus paused chest compressions during cardiac arrest in the emergency department | United States | Urban | Minneapolis, MN | To determine the frequency and duration of interruptions in CPR during the intubation procedure, and compare first attempt success when chest compressions are continuous versus interrupted. | Retrospective observational study | 169 |
| 2021 | Full Text | Measuring non-technical skills during prehospital advanced cardiac life support: A pilot study | Europe | Urban | Leuven, Belgium | To analyze non-technical skills of mobile medical teams during out-of-hospital cardiac arrests (OHCA) using the validated Team Emergency Assessment Measure (TEAM) tool. To research the correlation between non-technical skills and patient outcome. | Prospective observational study | 114 |
| 1992 | Full Text | Video recording of cardiac arrest management: an aid to training and audit | Europe | Urban | Cardiff, UK | To assess management of true cardiac arrests utilizing a video camera. | Prospective observational study | 10 |
| 1999 | Full Text | Leadership of resuscitation teams: ‚ÄòLighthouse Leadership‚Äô | Europe | Urban | Plymouth, UK | To determine the relationship between leadership behavior, team dynamics, and task performance in cardiopulmonary arrest resuscitations. | Prospective observational study | 20 |
| 2010 | Full Text | Cardiopulmonary resuscitation interruptions with use of a load-distributing band device during emergency department cardiac arrest | Asia | Urban | Singapore, Singapore | To measure no-flow time and no-flow ratio before and after an emergency department switched from manual to load-distributing band mechanical cardiopulmonary resuscitation (CPR) device | Before-and-after | 67 |
| 2010 | Full Text | Improving cardiopulmonary resuscitation in the emergency department by real-time video recording and regular feedback learning | Asia | Urban | Wuhan City, China | To test the hypothesis that video recording associated with regular feedback learning can rapidly and efficiently improve CPR quality provided by an in-hospital emergency team | Prospective observational study | 45 |
| 2017 | Full Text | Ultrasound use during cardiopulmonary resuscitation is associated with delays in chest compressions | United States | Urban | Baltimore, MD | To determine the impact of POCUS during cardiac arrest resuscitation on the duration of pulse checks in emergency department (ED) patients | Before-and-after | 23 |
| 2013 | Full Text | The Validity of Cardiopulmonary Resuscitation Skills in the Emergency Department Using Video-Assisted Surveillance: An Iranian Experience | Asia | Urban | Tehran, Iran | To evaluate the quality of CPR procedures performed in Tehran‚Äôs Rasool-e-Akram Hospital, the first Emergency Medicine academic center in Iran, using a videotaped real-life (actual) CPR technique, with the aim of pointing out the defects and shortcomings in this regard. | Prospective observational study | 50 |
| 2018 | Full Text | Emergency department use of a mechanical chest compression device frequently causes unanticipated interruptions in cardiopulmonary resuscitation | United States | Urban | Oakland, CA | Letter to the Editor | Letters and Replies | 86 |
| 2017 | Full Text | Introduction of paramedic led Echo in Life Support into the pre-hospital environment: The PUCA study | Europe | Urban | Edinburgh, UK | To perform satisfactory pre-hospital Echo in Life Support during the 10-s pulse check window and to see if pre-hospital ELS adversely affects the delivery of cardiac arrest (elongation of hands off the chest time, quality of life support) | Prospective observational study | 45 |
| 2023 | Full Text | Impact of Ultrasonography on Chest Compression Fraction and Survival in Patients with Out-of-hospital Cardiac Arrest | Asia | Urban | Taipei, Taiwan | Investigate the impact of ultrasound on chest compression fraction (CCF) and patient survival | Retrospective observational study | 236 |
| 2015 | Full Text | Evaluating the Quality of Cardiopulmonary Resuscitation in the Emergency Department by Real-Time Video Recording System | Asia | Urban | Shanghai, China | To evaluate the quality of chest compressions and comparing the effects of manual-CPR and MCC-CPR | Retrospective observational study | 112 |
| 2012 | Full Text | Video recording and feedback of resuscitation | Asia | Urban | Taipei City, Taiwan |  | Letters and Replies |  |
| 2012 | Full Text | Reply to Letter: Video Recording and Feedback of Resuscitation | Asia | Urban | Wuhan City, Hubei Province, China | | Letters and Replies |  |
| 2014 | Full Text | Medical students do not adversely affect the quality of cardiopulmonary resuscitation for ED patients | Asia | Urban | Wuhan, China | To determine whether the quality of cardiopulmonary resuscitation (CPR) was altered by the involvement of medical students in our emergency department (ED) | Retrospective observational study | 66 |
| 2019 | Full Text | Sudden cardiac arrest in sports: a video analysis | Europe | Urban | Oslo, Norway | To investigate the mechanisms and characteristics of SCA in athletes through a detailed video analysis. | Case series | 35 |
| 2018 | Full Text | How much experience do rescuers require to achieve successful tracheal intubation during cardiopulmonary resuscitation | Asia | Urban | Seoul, Republic of Korea | To evaluate how much experience with endotracheal intubation (ETI) is required for rescuers to perform successful ETI quickly without complications including serious chest compression interruption (interruption time <10s) or esophageal intubation during CPR | Retrospective observational study | 93 |
| 2022 | Full Text | Application of the Team Emergency Assessment Measure for Prehospital Cardiopulmonary Resuscitation | Asia |  | Bucheon, Korea Hwaseong, Korea Seoul, Korea Cheonan, Korea Goyang, Korea Guri, Korea Gwangju, Korea Incheon, Korea | To apply the Team Emergency Assessment Measure (TEAM) tool - which is considered to be the most promising approach in CPR situations among the currently known teamwork evaluation tools - to prehospital situations and confirm its suitability in prehospital CPR performed by the EMS. | Retrospective observational study | 67 |
| 2007 | Full Text | Video-recording and time-motion analyses of manual versus mechanical cardiopulmonary resuscitation during ambulance transport | Asia | Urban | Taipei, Taiwan | to identify operator- and ambulance-related factors affecting CPR quality during ambulance transport; and to assess the effectiveness of mechanical CPR device in such environment. | Prospective observational study | 19 |
| 2012 | Full Text | Accuracy of the cardiopulmonary resuscitation registry in an emergency department | Asia | Urban | Seoul, South Korea | To assess the accuracy of the CPR registry using CCTV-based data and to evaluate whether CCTV review can be used in describing CPR events. | Prospective observational study | 40 |
| 2020 | Full Text | Comparison of the effects of audio-instructed and video-instructed dispatcher-assisted cardiopulmonary resuscitation on resuscitation outcomes after out-of-hospital cardiac arrest | Asia | Urban | Seoul, South Korea | To describe the characteristics of OHCA provided with video-instructed and audio-instructed DA-CPR and to compare the real-world survival outcomes of OHCA patients according to DA-CPR method | Retrospective observational study | 1720 |
| 2021 | Full Text | Live video from bystanders‚Äô smartphones to improve cardiopulmonary resuscitation | Europe | Urban | Copenhagen, Denmark | To investigate whether live video streaming from the bystander‚Äôs smartphone to a medical dispatcher can improve the quality of bystander cardiopulmonary resuscitation (CPR) in out-of-hospital cardiac arrest (OHCA) | Retrospective observational study | 52 |
| 2015 | Full Text | Challenges in out-of-hospital cardiac arrest- A study combining closed-circuit television (CCTV) and medical emergency calls | Europe | Urban | Copenhagen, Denmark | To explore the challenges in recognition and initial treatment of OHCA by using CCTV recordings combined with audio recordings from emergency calls | Retrospective observational study | 21 |
| 2021 | Full Text | Immediate Bystander Cardiopulmonary Resuscitation to Sudden Cardiac Arrest During Sports is Associated with Improved Survival- a video Analysis | Europe | Other: NA | Amsterdam, Netherlands | To determine the effect of rapid bystander CPR to SCA during sports by searching for and analyzing videos of these SCA/SCD events from the internet. | Retrospective observational study | 29 |
| 2021 | Full Text | Impact of video-recording on patient outcome and data collection in out-of-hospital cardiac arrests | Europe | Urban | Leuven, Belgium | To compare the data registration of the medical record with the data from the video recorded resuscitation to study the impact of video recording during resuscitation on the outcome, and outcomes of OHCA before and after recording. | Prospective observational study | 129 |
| 2018 | Full Text | Point-of-care ultrasound use in patients with cardiac arrest is associated prolonged cardiopulmonary resuscitation pauses: A prospective cohort study | United States | Urban | Oakland, CA | To evaluate if point-of-care ultrasound use in cardiac arrest is associated with increased CPR pause duration | Prospective observational study | 24 |
| 2013 | Full Text | Obstacles Delaying the Prompt Deployment of Piston-Type Mechanical Cardiopulmonary Resuscitation Devices During Emergency Department Resuscitation: A Video-Recording and Time-Motion Study | Asia | Urban | Taipei, Taiwan | To identify the timeliness of the overall and of each essential step in the deployment of a piston-type MD during emergency department (ED) resuscitation, and to identify factors associated with delayed MD deployment by video recordings. | Retrospective observational study | 37 |
| 2015 | Full Text | The combined use of mechanical CPR and a carry sheet to maintain quality resuscitation in out-of-hospital cardiac arrest patients during extrication and transport | Europe | Urban | Edinburgh, UK | To evaluate the introduction of a mechanical CPR device combined with a patient carry sheet into a pre-hospital cardiac arrest response team and objectively assess the quality of resuscitation in refractory OHCA patients transported to hospital with on going CPR | Prospective observational study | 119 |
| 1996 | Full Text | Comparison of cardiopulmonary resuscitation techniques using video camera recordings | Europe | Urban | Portsmouth, England | To use video recordings to compare the performance of resuscitation teams in relation to their previous training in cardiac resuscitation. | Retrospective observational study | 101 |
| 2021 | Full Text | Assessment of chest compression interruptions during advanced cardiac life support | Europe | Urban | Leuven, Belgium | To identify potentially avoidable factors responsible for chest compression interruptions and to evaluate the influence of chest compression fraction on achieving return of spontaneous circulation and survival to hospital discharge | Prospective observational study | 206 |
| 2013 | Full Text | Feasibility of the video-laryngoscope (GlideScope¬Æ) for endotracheal intubation during uninterrupted chest compressions in actual advanced life support: A clinical observational study in an urban emergency department | Asia | Urban | Seoul, Republic of Korea | To evaluate whether successful ETI using a VL can be performed easily without failed intubation or complications, as well as a minimal frequency and duration of interruptions to chest compressions. | Prospective observational study | 71 |
| 2022 | Full Text | Timing and Identification of the Cause and Treatment of a Cardiac Arrest: A Potential Survival Benefit | Europe | Urban | Leuven, Belgium | First, to analyze how emergency physicians performed an etiologic evaluation. Second, to evaluate adherence to the ACLS algorithm in the search for the cause of arrest. Third, to investigate the influence of the discovery of presumable etiology on achieving ROSC. | Prospective observational study | 139 |
| 2019 | Full Text | Emergency Department Management of Out-of-Hospital Laryngeal Tubes | United States | Urban | Minneapolis, Minnesota | To describe ED airway management techniques, success, and complications of patients arriving at the ED with an EMS-placed laryngeal tube | Retrospective observational study | 486 |
| 2020 | Full Text | Association of ultrasound-related interruption during cardiopulmonary resuscitation with adult cardiac arrest outcomes: A video-reviewed retrospective study | United States | Urban | Brooklyn, NY | To determine the association of focused transthoracic echocardiography (ECHO) related interruption during cardiopulmonary resuscitation (CPR) with patient outcomes in the Emergency Department (ED) | Retrospective observational study | 210 |
| 2020 | Full Text | Minimizing Pulse Check Duration Through Educational Video Review | United States | Urban | Washington, D.C. | To investigate if CPR video review with feedback and education improve pulse check times with POCUS use | Cohort study | 70 |
| 2023 | Full Text | Emergency Medical Services Handoff of Patients in Cardiac Arrest in the Emergency Department: A Retrospective Video Review Study of Duration and Details of Handoff | United States | Urban | Manhasset, NY | To evaluate the duration and frequency of communication between EMS and ED staff during handoff and the subsequent time to critical cardiac care (rhythm determination, defibrillation) using cardiac arrest video review. | Retrospective observational study | 95 |
| 2020 | Full Text | Mechanical, Team-Focused, Video-Reviewed Cardiopulmonary Resuscitation Improves Return of Spontaneous Circulation After Emergency Department Implementation | United States | Urban | Manhasset, NY | To determine if mechanical, team-focused, video-reviewed cardiopulmonary resuscitation improved ROSC and to assess other process and outcome measures important in the care of cardiac arrest patients | Retrospective observational study | 248 |
| 2021 | Full Text | Video case review for quality improvement during cardiac arrest resuscitation in the emergency department | United States | Urban | Washington DC | To improve compliance with AHA quality metrics. Hypothesis being adult CPR video review would improve pulse check times in OHCA | Prospective observational study | 94 |
| 2018 | Full Text | Implementation of the Cardiac Arrest Sonographic Assessment (CASA) protocol for patients with cardiac arrest is associated with shorter CPR pulse checks | United States | Urban | Oakland, California | To determine whether implementation of the "Cardiac Arrest Sonographic Assessment" (CASA) protocol reduces the duration of interruptions in CPR during resuscitation of cardiac arrest (CA) compared to pre-intervention period | Before-and-after | 83 |

We are committed to sharing the raw data from our review upon reasonable request.
